# Supplementary material for: ProteinShader: illustrative rendering of macromolecules
Source: BMC Struct Biol. 2009 Mar 30;9:19. doi: 10.1186/1472-6807-9-19 (PMC2672931; doi:10.1186/1472-6807-9-19)
Supplement: Additional file 1 — ProteinShader program without source code. This compressed file contains the complete ProteinShader program including associated libraries, but no source code. A README.txt file gives an overview of the ProteinShader distribution, and the index.html file in the help subdirectory has directions on getting started with the program as well as a set of tutorials. [file 1472-6807-9-19-S1.zip › ProteinShader-beta-0_9_4-binary/help/api/org/proteinshader/structure/visitor/BondGeneratorVisitor.html]

BondGeneratorVisitor (ProteinShader API)


|  |  |  |
| --- | --- | --- |
| **Overview** \| **Package** \| **Class** \| **Use** \| **Tree** \| **Deprecated** \| **Index** \| **Help** | |  |
| **PREV CLASS**   **NEXT CLASS** | **FRAMES**    **NO FRAMES**     **All Classes** |  |
| SUMMARY: NESTED \| FIELD \| CONSTR \| METHOD | DETAIL: FIELD \| CONSTR \| METHOD |  |


---


## org.proteinshader.structure.visitor Class BondGeneratorVisitor

```
java.lang.Object
  org.proteinshader.structure.visitor.Visitor
      org.proteinshader.structure.visitor.BondGeneratorVisitor
```

---

``` public class BondGeneratorVisitor extends Visitor ```

Generates standard Bonds for AminoAcids and Waters.
Peptide Bonds, intra-AminoAcid Bonds, and the oxygen-hydrogen Bonds of
Water are generated after calculating which Atoms are close enough to
form a Bond. The algorithm for Bond prediction is given in the
BondPredictor class.
  
  
 **Usage**   
  
 `structure.accept( new BondGeneratorVisitor() );` 
  
  
will generate AminoAcid and Water Bonds on the Structure, while
  
  
 `Visitor visitor = new BondGeneratorVisitor();   
visitor.setMode( ResidueMode.AMINO_ACIDS );   
structure.accept( visitor );` 
  
  
would only generate Bonds for the AminoAcids.
  
  
  **Note on Exception Handling**    
  
Currently, the VisitorException in the visit() method signatures is
only there to satisfy the Visitor API (other Visitors such as the
SFWriterVisitor do need an exception in the visit() method
declaration).

---

**Constructor Summary**

`BondGeneratorVisitor()`
  
          Constructs a BondGeneratorVisitor.


| **Method Summary** | |
| --- | --- |
| `void` | `setAABondVisibility(VisibilityEnum visibility)`             Determines what visibility status the Bond generator will use when generating Bonds for AminoAcids. |
| `void` | `setMinBondLength(double minBondLength)`             Sets the minimum Bond length value used for Bond prediction. |
| `void` | `setTolerance(double tolerance)`             Sets the tolerance value used for Bond prediction. |
| `void` | `setWaterBondVisibility(VisibilityEnum visibility)`             Determines what visibility status the Bond generator use when generating Bonds for Waters. |
| `void` | `visit(AminoAcid aminoAcid)`             Uses Atom distances to generate Bonds within the AminoAcid, and also uses the memory of the last carbonyl carbon seen to check for a possible peptide Bond. |
| `void` | `visit(Atom atom)`             Checks if the Atom is a possible Bond destination Atom for any previously seen Atoms of the same Residue, and then adds the Atom to the list of Atoms to remember. |
| `void` | `visit(Bond bond)`             NO OPERATION: This Visitor method is not needed by the bond generator. |
| `void` | `visit(Chain chain)`             Generates all standard Bonds (AminoAcids and Waters) for the Chain. |
| `void` | `visit(Heterogen heterogen)`             NO OPERATION: This method is not needed because non-water heterogen bonds are given explicitly in the PDB structure entry. |
| `void` | `visit(Model model)`             Generates all standard Bonds (AminoAcids and Waters) for the Model. |
| `void` | `visit(Structure structure)`             Generates all standard Bonds (AminoAcids and Waters) for the Structure. |
| `void` | `visit(Water water)`             If the Water includes hydrogen atoms, then Bonds will be created between the oxygen and each hydrogen (after checking that the Bond lengths are reasonable). |

**Methods inherited from class org.proteinshader.structure.visitor.Visitor**

`clear, includeAAHetAndWater, setDebug, setMode, setMode, setMode, setMode, setMode, setMode, setVisitSegments, visit, visit, visit, visit`

**Methods inherited from class java.lang.Object**

`clone, equals, finalize, getClass, hashCode, notify, notifyAll, toString, wait, wait, wait`

**Constructor Detail**

### BondGeneratorVisitor

```
public BondGeneratorVisitor()
```

:   Constructs a BondGeneratorVisitor. The Bonds are set to
    Visiblity.OPAQUE by default.


**Method Detail**

### setAABondVisibility

```
public void setAABondVisibility(VisibilityEnum visibility)
```

:   Determines what visibility status the Bond generator will use when
    generating Bonds for AminoAcids. If null is given as an argument,
    the visiblity will be set to VisibilityEnum.INVISIBLE.

    :   **Parameters:**: `visibility` - the visibility status for AminoAcid Bonds.

---


### setWaterBondVisibility

```
public void setWaterBondVisibility(VisibilityEnum visibility)
```

:   Determines what visibility status the Bond generator use when
    generating Bonds for Waters. If null is given as an argument, the
    visiblity will be set to VisibilityEnum.INVISIBLE.

    :   **Parameters:**: `visibility` - the visibility status for Water Bonds.

---


### setTolerance

```
public void setTolerance(double tolerance)
```

:   Sets the tolerance value used for Bond prediction.

    :   **Parameters:**: `tolerance` - tolerance value in Angstroms.

---


### setMinBondLength

```
public void setMinBondLength(double minBondLength)
```

:   Sets the minimum Bond length value used for Bond prediction.

    :   **Parameters:**: `minBondLength` - minimum Bond length in Angstroms.

---


### visit

```
public void visit(Structure structure)
           throws VisitorException
```

:   Generates all standard Bonds (AminoAcids and Waters) for the
    Structure.

    :   **Overrides:**: `visit` in class `Visitor`
    :   **Parameters:**: `structure` - the Structure to generate Bonds for. **Throws:**: `VisitorException` - if an error occurs while generating Bonds.

---


### visit

```
public void visit(Model model)
           throws VisitorException
```

:   Generates all standard Bonds (AminoAcids and Waters) for the
    Model.

    :   **Overrides:**: `visit` in class `Visitor`
    :   **Parameters:**: `model` - the Model to generate Bonds for. **Throws:**: `VisitorException` - if an error occurs while generating Bonds.

---


### visit

```
public void visit(Chain chain)
           throws VisitorException
```

:   Generates all standard Bonds (AminoAcids and Waters) for the
    Chain.

---


### visit

```
public void visit(AminoAcid aminoAcid)
           throws VisitorException
```

:   Uses Atom distances to generate Bonds within the AminoAcid, and
    also uses the memory of the last carbonyl carbon seen to check for
    a possible peptide Bond.

---


### visit

```
public void visit(Heterogen heterogen)
           throws VisitorException
```

:   NO OPERATION: This method is not needed because non-water
    heterogen bonds are given explicitly in the PDB structure entry.

    :   **Overrides:**: `visit` in class `Visitor`
    :   **Parameters:**: `heterogen` - the Heterogen to do nothing with. **Throws:**: `VisitorException` - will not happen.

---


### visit

```
public void visit(Water water)
           throws VisitorException
```

:   If the Water includes hydrogen atoms, then Bonds will be created
    between the oxygen and each hydrogen (after checking that the Bond
    lengths are reasonable).

---


### visit

```
public void visit(Atom atom)
           throws VisitorException
```

:   Checks if the Atom is a possible Bond destination Atom for any
    previously seen Atoms of the same Residue, and then adds the Atom
    to the list of Atoms to remember.

---


### visit

```
public void visit(Bond bond)
           throws VisitorException
```

:   NO OPERATION: This Visitor method is not needed by the bond
    generator.

    :   **Overrides:**: `visit` in class `Visitor`
    :   **Parameters:**: `bond` - the Bond to do nothing with. **Throws:**: `VisitorException` - will not happen.


---


|  |  |  |
| --- | --- | --- |
| **Overview** \| **Package** \| **Class** \| **Use** \| **Tree** \| **Deprecated** \| **Index** \| **Help** | |  |
| **PREV CLASS**   **NEXT CLASS** | **FRAMES**    **NO FRAMES**     **All Classes** |  |
| SUMMARY: NESTED \| FIELD \| CONSTR \| METHOD | DETAIL: FIELD \| CONSTR \| METHOD |  |


---

# *Copyright © 2007-2008*
